# Genome-wide characterization of DNA methyltransferase family genes implies *GhDMT6* improving tolerance of salt and drought on cotton

**DOI:** 10.1186/s12870-024-04985-x

**Published:** 2024-04-23

**Authors:** Xiaomin Yang, Zhigang Bai, Yunxin He, Ning Wang, Liangqing Sun, Yongqi Li, Zujun Yin, Xiaoge Wang, Binglei Zhang, Mingge Han, Xuke Lu, Xiugui Chen, Delong Wang, Junjuan Wang, Shuai Wang, Lixue Guo, Chao Chen, Keyun Feng, Wuwei Ye

**Affiliations:** 1 Institute of Cotton Research of Chinese Academy of Agricultural Sciences / Research Base, State Key Laboratory of Cotton Bio-breeding and Integrated Utilization, School of Agricultural Sciences, Zhengzhou University, Anyang, Henan 455000 China; 2 Cash Crop Research Institute of Jiangxi Province, Jiujiang, Jiangxi 332105 China; 3 Hunan Institute of Cotton Science, Changde, Hunan 415101 China; 4 Institute of Crop Sciences, Gansu Academy of Agricultural Sciences, Lanzhou, Gansu 730070 China

**Keywords:** *C5-MTase*, *Gossypium raimondii*, *Gossypium arboreum*, *Gossypium hirsutum*, Abiotic stress

## Abstract

**Background:**

DNA methylation is an important epigenetic mode of genomic DNA modification and plays a vital role in maintaining epigenetic content and regulating gene expression. *Cytosine-5 DNA methyltransferase* (*C5-MTase)* are the key enzymes in the process of DNA methylation. However, there is no systematic analysis of the *C5-MTase* in cotton so far, and the function of DNMT2 genes has not been studied.

**Methods:**

In this study, the whole genome of cotton *C5-MTase* coding genes was identified and analyzed using a bioinformatics method based on information from the cotton genome, and the function of *GhDMT6* was further validated by VIGS experiments and subcellular localization analysis.

**Results:**

33 *C5-MTases* were identified from three cotton genomes, and were divided into four subfamilies by systematic evolutionary analysis. After the protein domain alignment of C5-MTases in cotton, 6 highly conserved motifs were found in the C-terminus of 33 proteins involved in methylation modification, which indicated that C5-MTases had a basic catalytic methylation function. These proteins were divided into four classes based on the N-terminal difference, of which DNMT2 lacks the N-terminal regulatory domain. The expression of *C5-MTases* in different parts of cotton was different under different stress treatments, which indicated the functional diversity of cotton *C5-MTase* gene family. Among the *C5-MTases*, the *GhDMT6* had a obvious up-regulated expression. After silencing *GhDMT6* with VIGS, the phenotype of cotton seedlings under different stress treatments showed a significant difference. Compared with cotton seedlings that did not silence *GhDMT6*, cotton seedlings silencing *GhDMT6* showed significant stress resistance.

**Conclusion:**

The results show that *C5-MTases* plays an important role in cotton stress response, which is beneficial to further explore the function of DNMT2 subfamily genes.

**Supplementary Information:**

The online version contains supplementary material available at 10.1186/s12870-024-04985-x.

## Background

DNA methylation is the process of transferring a methyl (-CH_3_) group to a specific base of a DNA molecule and is catalyzed by DNA methyltransferase, with S-adenosine methionine (SAM) as a methyl donor [1]. It widely occurs in the epigenetics of bacteria, plants, and animals and is involved in transposons, the suppression of gene silencing, genomic imprinting, X chromosome inactivation, cell differentiation and embryo development [2–5]. DNA methylation is a dynamic process. When plants are subjected to stress, DNA methylation breaks through the inherent stability and limitation of their genome and makes a rapid response to adversity [6]. This induces the expression of some genes associated with stress to maintain plant growth, development and evolutionary process. Therefore, epigenetic modification precedes genomic evolution in response to adversity, and DNA methylation is considered the molecular response mechanism of plants in the face of adverse stress.

DNA methylation mostly take place in CHG at carbon 5 in cytosine (C5) [7] and primarily occurs in symmetric sequence CHG but also in CHG and CHH (H = A, C or T) sequences [5, 8]. There are two DNA methylation methods in plants: maintenance methylation and *denovo* methylation [9]. The former means that double-stranded DNA molecule through semi-reserved replication, which is distinguished by whether it is methylated. Parental methylation patterns are passed to the offspring [10]. The latter is a type of DNA methylation that happens when different *C5-Mtases* catalyze two strands of DNA without methylation. *C5-Mtases* in plants cotain four categories, which respectively are methyltransferase (MET), chromo methylase (CMT), domains rearranged methylase (DRM), and (DNA methyltransferase homologue 2)DNMT2 [11, 12]. METs are mainly used in methylation of the heterochromatin region of the CHG site of the symmetric sequence, which is an indispensable part of the methyltransferase [13]. CMTs are a special type of C5-Mtases that maintain methylation at CHG and CHH sites, and play a role in stabilizing the heterochromatin state of the genome [14]. DRM is homologous to the Dnmt3 of animals, and the function of them is to catalyze the methylation of cytosine and to maintain the cytosine methylation of non-CHG sites under the guidance of RNA [15, 16]. In previous study, after knockout of OsDRM2 gene, de novo methylation defect appeared in rice, which affected the DNA methylation level of rice genome, and finally led to abnormal reproductive and vegetative growth of rice [17]. The proteins encoded by the plant DNMT2 subfamily are very similar to those of mice, bacteria and yeast, and researches has shown that the DNMT2 subfamily genes may play a role in RNA methylation [18].

Abiotic stress can threaten the vegetative and reproductive growth of plants, accelerate the growth period and reduce the yield and quality of crops. Many studies have reported that the methylation level of plants also changes in response to abiotic stress [19, 20]. When subjected to different stresses, different DNA methylation in plant will active in the genome, which can regulate the expression of different stress response genes, thus opening up different regulatory pathways and improving plant tolerance [21]. DNA methylation is a very important regulatory pathway throughout the life cycle of organisms and plays a crucial role in the process of biological evolution, which is involved not only in growth and development but also in secondary metabolism [22–25]. DNA methylation is involved in the regulation of biological clock, photoperiod, stress resistance, metabolism, growth and development in many plant processes, including *arabidopsis thaliana* [26], *solanaceae* [27], *legumes* [21], maize [28], *solanum lycopersicum* [29]. However, studies on DNA methylation of cotton, which is an very important cash crop, have not been reported.

With the rapid change of global climate, the plant of cotton have been severely tested, especially the salt stress and drought stress seriously affect the yield and fiber quality of cotton. Many studies have reported that there is a close relationship between methylation levels and stress resistance in plants [30, 31], and indicated that *C5-MTase* is a key factor triggering methylation and can directly affect methylation levels [32].

However, there has been no relevant identification of *C5-MTase* in the cotton genome, and there has been no relevant study on how *C5-MTase* functions in response to salt stress and drought stress in cotton. With the completion of cotton genome sequencing [33–35], *C5-MTase* in cotton can be analyzed by genome-wide identification. In addition, the transcriptional abundance of *GhDMT6* under salt stress and drought stress was analyzed. Our results provide a reference for the role of *C5-MTase* in abiotic stress resistance in cotton.

## Results

### Identification of C5-MTase family members

A total of 33 *C5-MTase* members were identified from the whole genome of cotton. Group A contained 9 *C5-MTases* and group D contained 8 *C5-MTases*, which were respectively named *GaDMT1*-*GaDMT9* and *GrDMT1*-*GrDMT8* according to their sequence on the chromosomes. Similarly, 16 *C5-MTases* were identified in the AD group, named *GhDMT1*-*GhDMT16*. Most of the *C5-MTases* are located on the chromosome in three cotton species. They consist of 344–1585 different amino acids, most of which contain 400–1000 amino acid residues. Because of the N-terminal difference in the gene structure, GaDMT3 contains up to 1585 amino acids, while GhDMT4 contains only 344 amino acids. The isoelectric point (PI) of C5-MTases ranges from 4.54 to 8.71. The predicted subcellular localization shows that most *C5-MTases* are located in the nucleus, but some are located in the outer chloroplast. Specially, *GaDMT7* and *GhDMT6* are located in the cell membrane (Table 1).

**Table 1:** Basic characteristics of *C5-MTases* in the cotton genome

gene name	Type	Locus ID	Location (chromosome)	Position (domain)	CDS (bp)	AA	PI	Predicted subcellular localization
GaDMT1	CMTc	Ga02G1310	Chr02:91041776-91047696-	442–816	2724	851	5.66	Nucleus
GaDMT2	DRM	Ga04G1492	Chr04:86372417-86376133-	511–630	4638	640	4.88	Chloroplast
GaDMT3	MET	Ga04G1657	Chr04:90780693-90787721-	1150–1579	1179	1585	5.51	Nucleus
GaDMT4	CMTd	Ga07G0535	Chr07:5708914–5,715,570 +	499–871	2082	907	5.09	Nucleus
GaDMT5	CMTa	Ga08G1800	Chr08:111527760-111536767-	589–729	2580	730	7.69	Nucleus
GaDMT6	CMTb	Ga08G1801	Chr08:111537181-111547908-	769–1145	1914	1182	8.64	Nucleus
GaDMT7	DNMT2	Ga08G2785	Chr08:127956353–127,959,973+	19–395	1992	398	6.41	Cell membrane, Endoplasmic reticulum
GaDMT8	DRM	Ga09G0343	Chr09:9182888–9,188,986+	569–687	1494	693	5.14	Chloroplast
GaDMT9	CMTd	Ga13G0623	Chr13:9274883-9284481-	431–799	2421	835	5.35	Cytoplasm
GrDMT1	CMTd	Gorai.001G052000	Chr01 : 4,942,493–4,949,993	507–867	2718	905	4.84	Nucleus
GrDMT2	CMTc	Gorai.002G216500	Chr02 : 56,627,700–56,634,321	454 ~ 812	2556	851	5.93	Nucleus
GrDMT3	CMTa	Gorai.004G180200	Chr04 : 49,072,088–49,079,643	587–945	2952	983	7.4	Nucleus
GrDMT4	CMTb	Gorai.004G180300	Chr04 : 49,082,203–49,092,215	789–1145	3558	1185	8.22	Nucleus
GrDMT5	DNMT2	Gorai.004G274400	Chr04 : 60,871,759–60,876,114	20–391	1221	406	6.05	Nucleus
GrDMT6	DRM	Gorai.006G031000	Chr06 : 8,112,196–8,117,039	513–627	1911	636	4.54	Chloroplast
GrDMT7	DRM	Gorai.012G062900	Chr12 : 8,876,628–8,881,084	526–640	1965	654	4.72	Chloroplast
GrDMT8	MET	Gorai.012G048000	Chr12 : 6,069,727–6,077,197	1144–1570	4734	1577	5.36	Nucleus
GhDMT1	DRM	CotAD_37635	At_chr06:11311066.11315214-	512–630	1911	636	4.8	Nucleus
GhDMT2	MET	CotAD_51709	At_chr9:63179674.63185543-	1076–1505	4536	1511	6.34	Nucleus
GhDMT3	DRM	CotAD_46796	At_chr9:65748738.65752451+	511–630	1923	640	4.85	Chloroplast
GhDMT4	CMTd	CotAD_10542	Dt_chr1:40856014.40859213-	1-308	1035	344	7.76	Nucleus
GhDMT5	CMTc	CotAD_49037	Dt_chr2:8190450.8196335-	496–781	2451	816	5.62	Nucleus
GhDMT6	DNMT2	CotAD_04205	Dt_chr5:13787674.13791315+	13–389	1206	401	5.82	Cell membrane, Endoplasmic reticulum
GhDMT7	DRM	CotAD_13275	Dt_chr6:40651384.40655909-	512–611	1878	625	4.72	Chloroplast
GhDMT8	CMTd	CotAD_24264	Dt_chr7:37416068.37421133+	230–632	2007	668	4.81	Nucleus
GhDMT9	CMTb	CotAD_00990	Dt_chr10: 6477855.6487242+	789–1157	3585	1194	8.51	Nucleus
GhDMT10	CMTa	CotAD_00992	Dt_chr10:6495924.6502242+	299–680	2094	697	6.75	Nucleus
GhDMT11	CMTc	CotAD_14980	scaffold39.1:602870.611908+	667–846	2646	881	6.94	Nucleus
GhDMT12	DRM	CotAD_18652	scaffold71.1:1581335.1591239-	547–666	2031	676	4.91	Cytoplasm
GhDMT13	CMTa	CotAD_41398	scaffold294.1:1053646.1057162-	319–500	1533	510	5.45	Cytoplasm
GhDMT14	CMTb	CotAD_41399	scaffold294.1:1063401:1074111-	780–1161	3597	1198	8.71	Nucleus
GhDMT15	MET	CotAD_46012	scaffold1041.1:189137.194922+	1112–1541	4644	1547	5.57	Nucleus
GhDMT16	CMTd	CotAD_40093	scaffold2005.1:29096.35764+	453–828	2595	864	5.02	Nucleus

### Chromosomal localization analysis

**Fig. 1:**
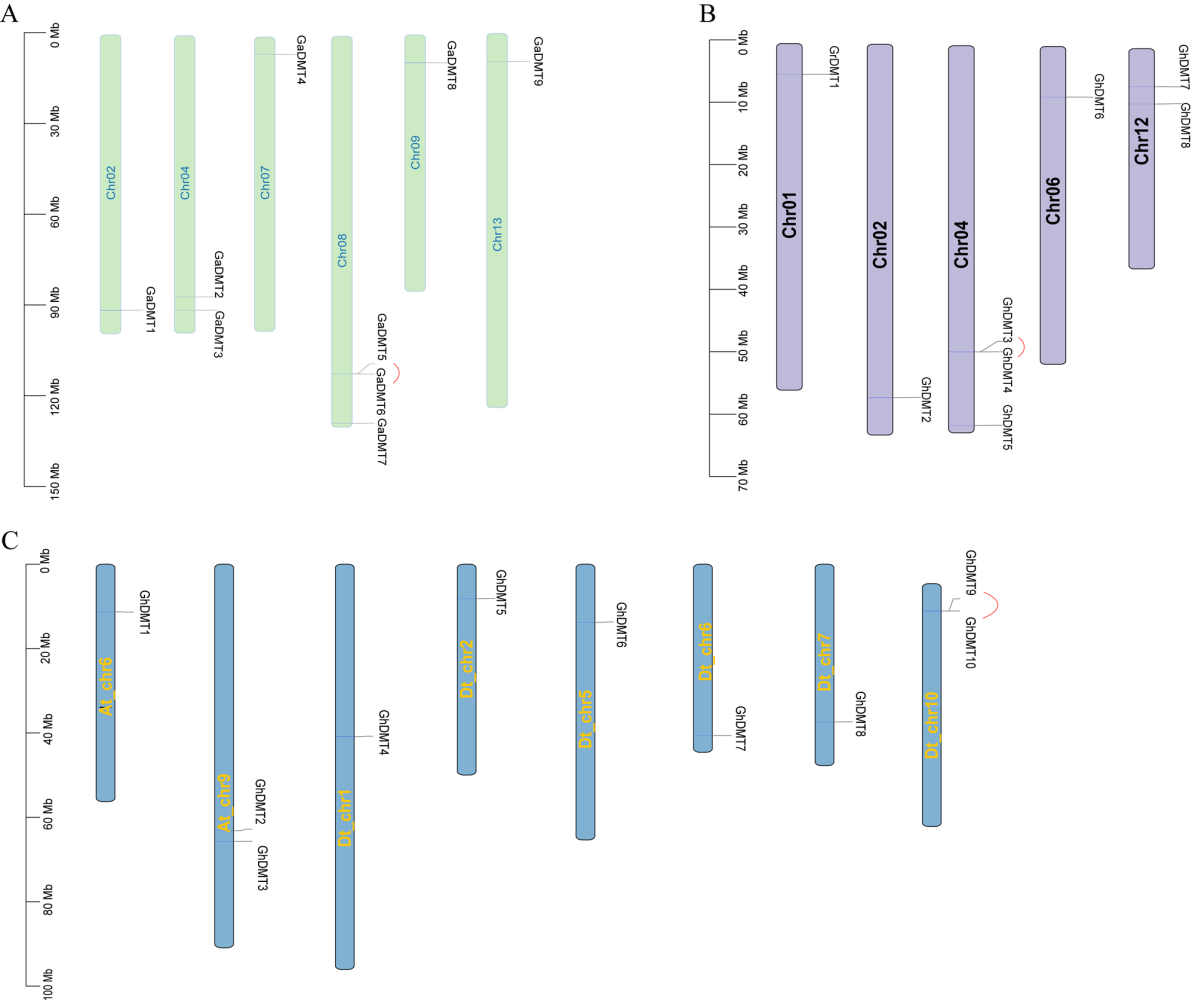
Chromosome distribution and syntenic analysis of *C5-MTases* from cotton. (**A**) Positions of *C5-MTase* gene family members on A group chromosomes. (**B**) Positions of *C5-MTase* gene family members on D group chromosomes. (**C**) Positions of *C5-MTase* gene family members on AD group chromosomes

The distribution map of the gene on the chromosome was constructed according to the gene locus information (Fig. 1). The 9 genes in group A were unevenly distributed on the 6 chromosomes of Chr02, Chr04, Chr07, Chr08, Chr09, and Chr13. Among them, Chr08 contained 3 genes on chromosome, which was the most one. *GaDMT5* and *GaDMT6* were a pair of tandem repeats (Fig. 1A). On the other hand, there were 8 genes in group D unevenly distributed on the 5 chromosomes of Chr01, Chr02, Chr04, Chr06, and Chr12. Among them, Chr06 has the most genes on chromosome, with 3 genes. *GrDMT7* and *GrDMT8* were a pair of tandem repeats (Fig. 1B). The AD group only mapped genes located on chromosomes (Fig. 1C). In the AD group, *GhDMT9* and *GhDMT10* were repeated in series. Among the 16 genes in AD group, 10 genes were located on chromosomes At_chr6、At_chr9、Dt_chr1、Dt_chr2、Dt_chr5、Dt_chr6、Dt_chr7、Dt_chr10, respectively, while another 6 genes were mapped to the scaffold.

### Conserved motifs and exon-intron analysis

**Fig. 2:**
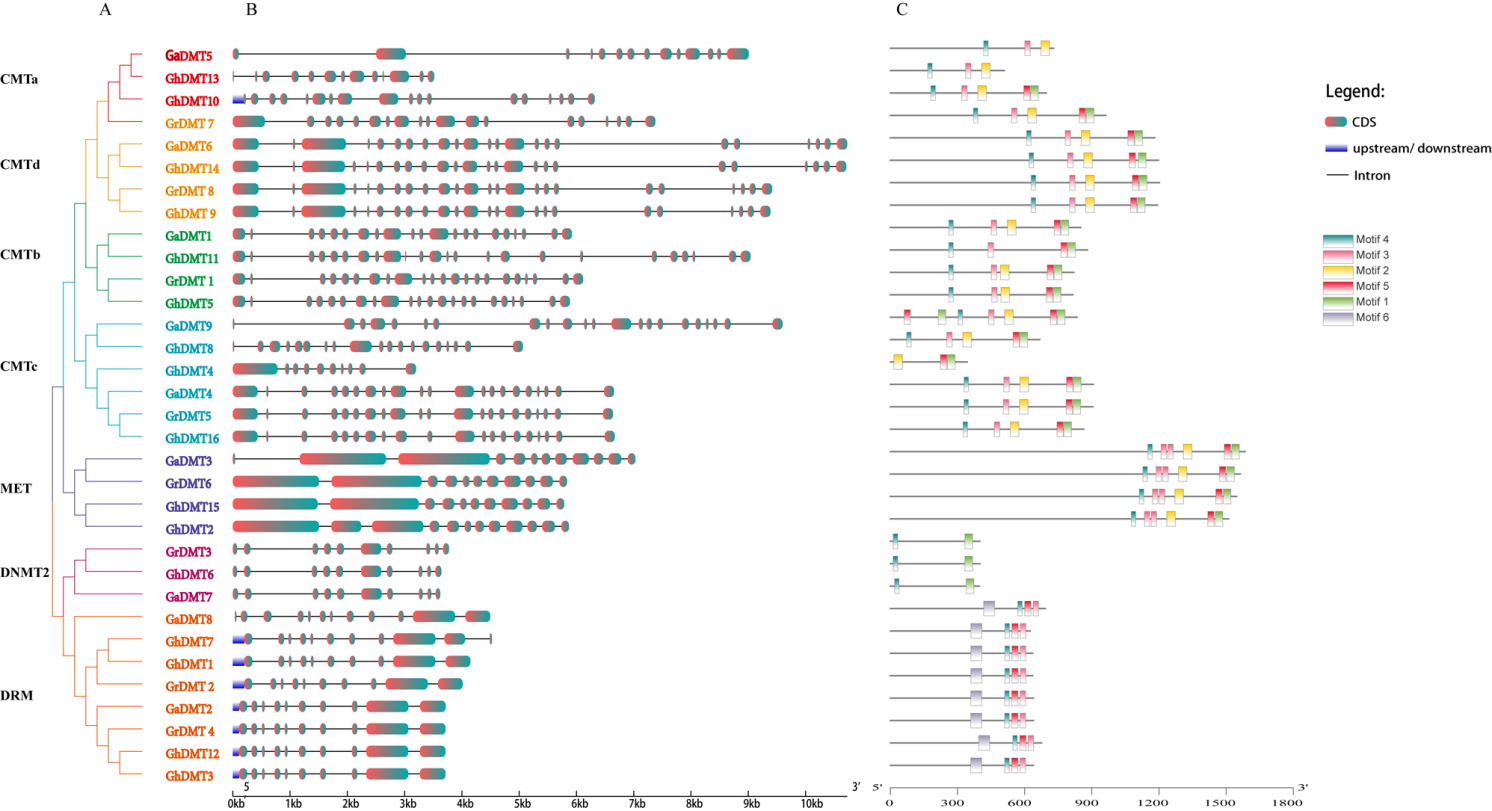
Conserved motifs and exon-intron analysis of *C5-MTases* in cotton. (**A**) Phylogenetic tree of *C5-MTases*. (**B**) Exon-intron structures of *C5-MTases*. (**C**) Conservative motif analysis

Visualized analysis of the *C5-MTase* gene structure and conserved motifs was shown in Fig. 2. Gene structure analysis is an important method in the study of genetic evolution. The number of introns and exons in *C5-MTase* family members in groups D, A, and AD were analyzed. The *C5-MTase*s structures for cotton were created (Fig. 2B). The results showed that the numbers of exons in different *C5-MTase*s in cotton varied greatly. *GrDMT8*, *GhDMT9*, *GhDMT11* and *GhDMT14* gene had the most exons, with 24 exons, while the *DNMT2*, *MET*, *CMTa*, *CMTb*, *CMTc*, *CMTd*, *DRM* subfamily contained 10, 11–12, 13–19, 23–24, 21–24, 10–21, 10–13 exons, respectively.

Conserved motifs of C5-MTases members in three cotton genomes were analyzed (Fig. 2C), and the protein sequences of 33 C5-MTases members were entered into the MEME website and set 6specific motifs. The C-terminal catalytic region had 6 highly conserved motifs. Among these motifs, motif 4 and motif 6 were used for SAM-binding sites, and motif 1, motif 2, motif 3, and motif 5 were the C5-Mtase functional catalytic sites. To be specific, motif 3 was the active site, motif 5 was the target cytosine binding site, motif 2 was the DNA neutralization region, motif 1 was the target sequence location identification area. In this study, it was found that all C5-MTases members contain motif 4 except GhDMT4. The motifs of C5-MTases members within the same subfamily were approximately same, however, it was quite different between different subfamilies, but. For example, almost all MET contained motifs 2, 3, 4 and 5, butthe vast majority of C5-MTases members in DRM included motifs 3, 4, 5 and 6, and the DNMT2 subfamily usually contained motif 4 and motif 1 only.

### Structural domain and promoter analysis

**Fig. 3:**
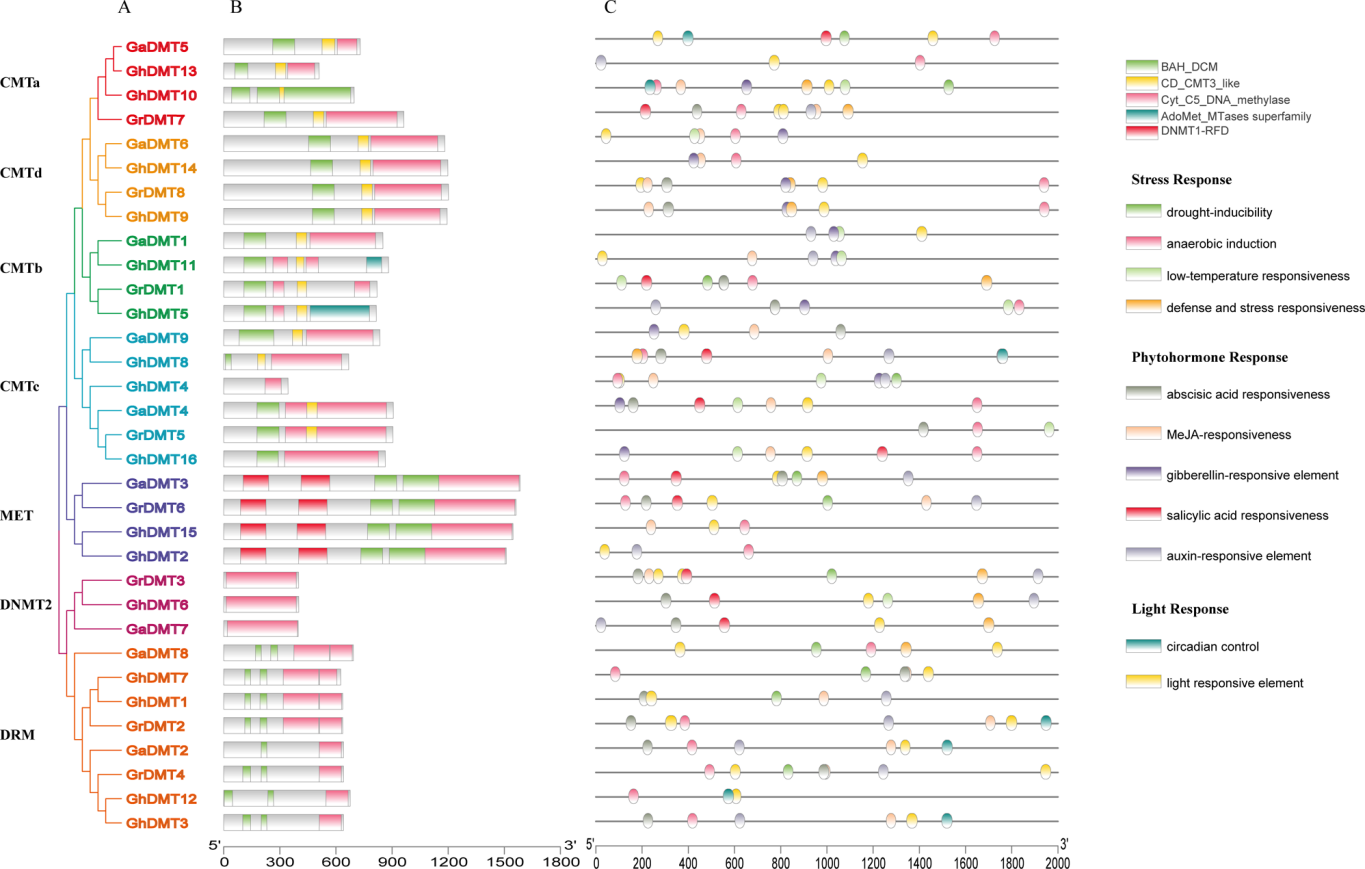
Structural domain and promoters of *C5-MTases* from three cotton species. (**A**) Phylogenetic tree of *C5-MTases*. (**B**) Structural domain of C5-MTases proteins. (**C**) Promoters of *C5-MTases*.**Collinear analysis**

The phylogenetic results of *C5-MTase* and the protein domain results of C5-MTase protein were analyzed (Fig. 3A). It was found that the same subfamily of genes had similar protein structure. After analyzing the domains of 33 C5-MTase proteins of cotton (Fig. 3B), it was found that all C5-MTase proteins contained Cyt-C5-DNA-methylase domains. The Cyt-C5-DNA-methylase domain was the conservative protein domain of the *C5-MTase* family. Genes contained different domains and were subdivided into different subfamilies. Genes contained similar domains and could be subdivided into the same subfamily. For example, the CMT subfamily contained the CD-CMT3-like domain except for *GhDMT16*, the MET subfamily contains 2 DNMT1-RFD domains and 2 BAH-DCM domains, and the DNMT2 subfamily contained only one Cyt-C5-DNA-methylase domain.

As a knob in the process of gene expression, promoters played a crucial role in plant growth and development. The promoters of 33 *C5-MTases* were analyzed in our study (Fig. 3C). The results showed that all *C5-MTases* promoters contained hormone response elements, but the distribution of these elements was not confirmed. It is also proved that that all the *C5-MTases* promoters contain one or more photo-responsive elements, which indicated that *C5-MTases* may be involved in plant circadian rhythm, photoperiod and other processes. Most of the *C5-MTases* promoters contained anaerobic induction elements and MeJA regulatory elements, and they also contained many different types of promoter elements.

**Fig. 4:**
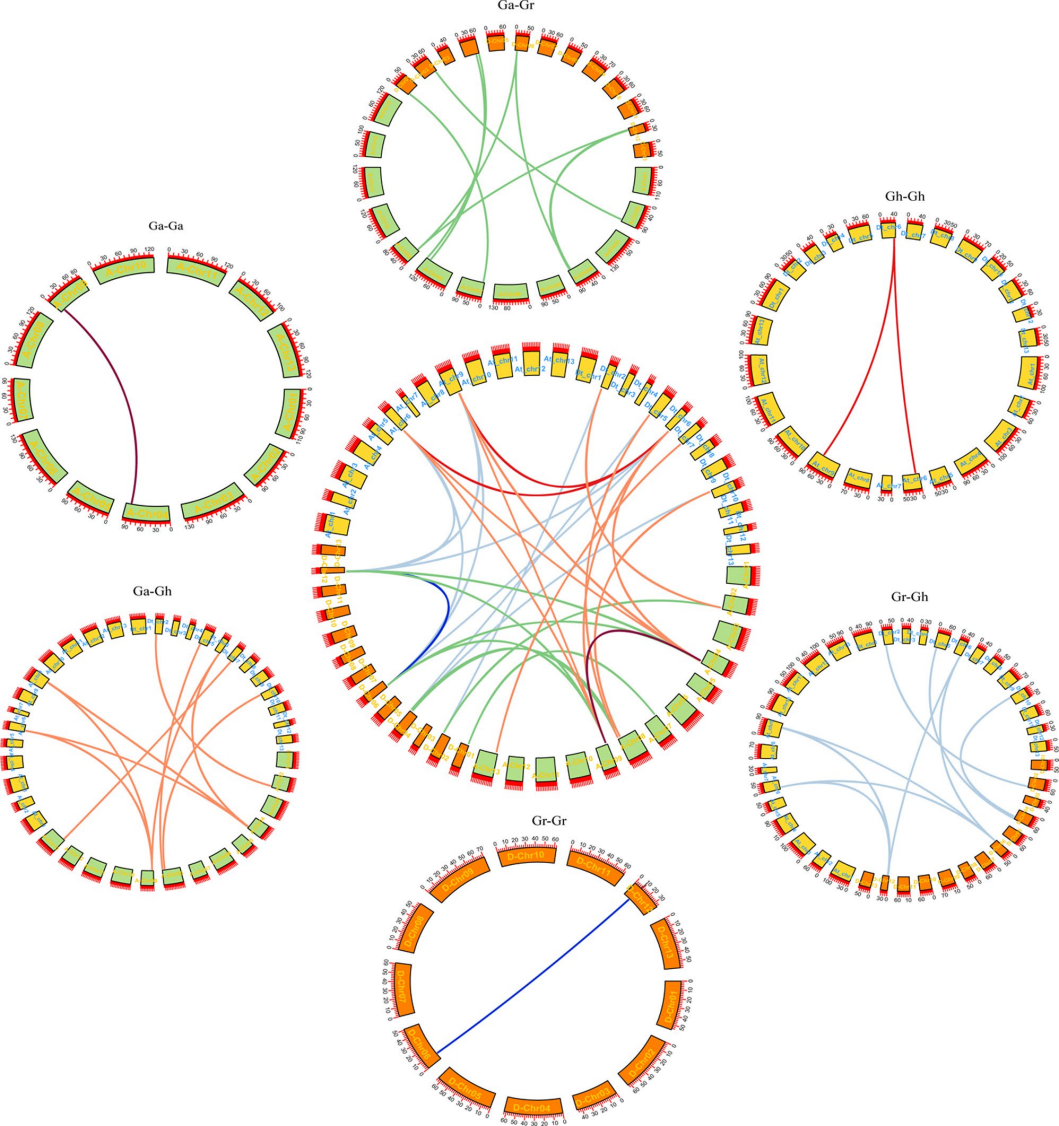
Syntenic relationship of *C5-MTases* duplicated gene pairs in A, D, and AD

In order to further investigate the evolutionary relationship between *G.arboreum*(A), *G.raimondii*(D) and *G.hirsutum*(AD) genomes, a collinear analysis between the two genomes was performed (Fig. 4). 56 linear gene pairs and 3 tandem repeat gene pairs were identified by collinearity analysis (Fig. 1). *GaDMT5* and *GaDMT6*, *GrDMT3* and *GrDMT4*, *GhDMT9* and *GhDMT10* were three tandem repeat gene pairs, respectively. Ga(A)-Ga(A), Gh(AD)-Gh(AD) and Gr(D)-Gr(D) had 1,10 and 1 co-linear gene pair, respectively. 18,9, and 17 linear gene pairs in Ga(A)-Gh(AD), Ga(A)-Gr(D), and Gh(AD)-Gr(D) were identified, respectively (supplementary table S3). A total of 44 genes were replicated genome-wide. Some of the *C5-MTase* genes in the AD group were mapped to scaffold, which caused that the gene pairs shown in the image were different from the actual ones. For example, a total of 10 homologous gene pairs were identified, but only 2 pairs were marked on the chromosome and 8 pairs were not.

**Fig. 5:**
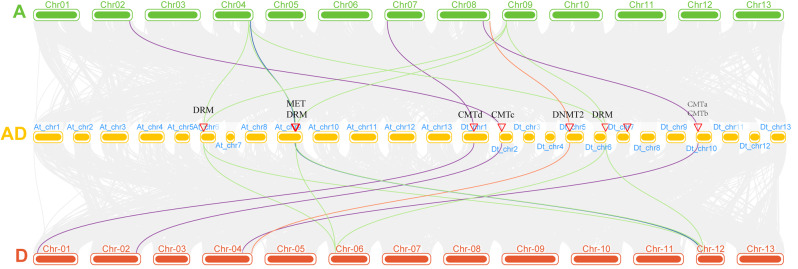
Multiple collinear analysis of *C5-MTase* genes from three cotton species. The CMT subfamily genes are linked by purple lines. The DRM subfamily genes are linked by green line. The MET subfamily genes are linked by blue line. The DNMT2 subfamily genes are linked by red lines

The cotton *C5-MTase* gene was multicollinearity and a collinear map was furtherly constructed between AD and A, D (Fig. 5). The finding showed that most of the *C5-MTases* were located on synlinear chromosomal segments, indicating that group A and group D had high similarity. CMT subfamily, DRM subfamily, MET subfamily, and DNMT2 subfamily all had orthologous genes in group A, group D, and group AD, indicating that the *C5-MTases* family was highly conserved in the course of evolution. The above characteristics were inherited in the process of doubling the hybridization between group A and group D to form the AD group. It is found that the CMTa subfamily has no orthologous genes. The reason was guessed that the function of this subfamily was similar to the other subfamily, which lead to it was lost or was replaced by other subfamily with evolution. All these results proved that the doubling of group A and group D to form group AD was an incomplete inheritance process.

### Selection pressure and interspecies evolution analysis

**Fig. 6:**
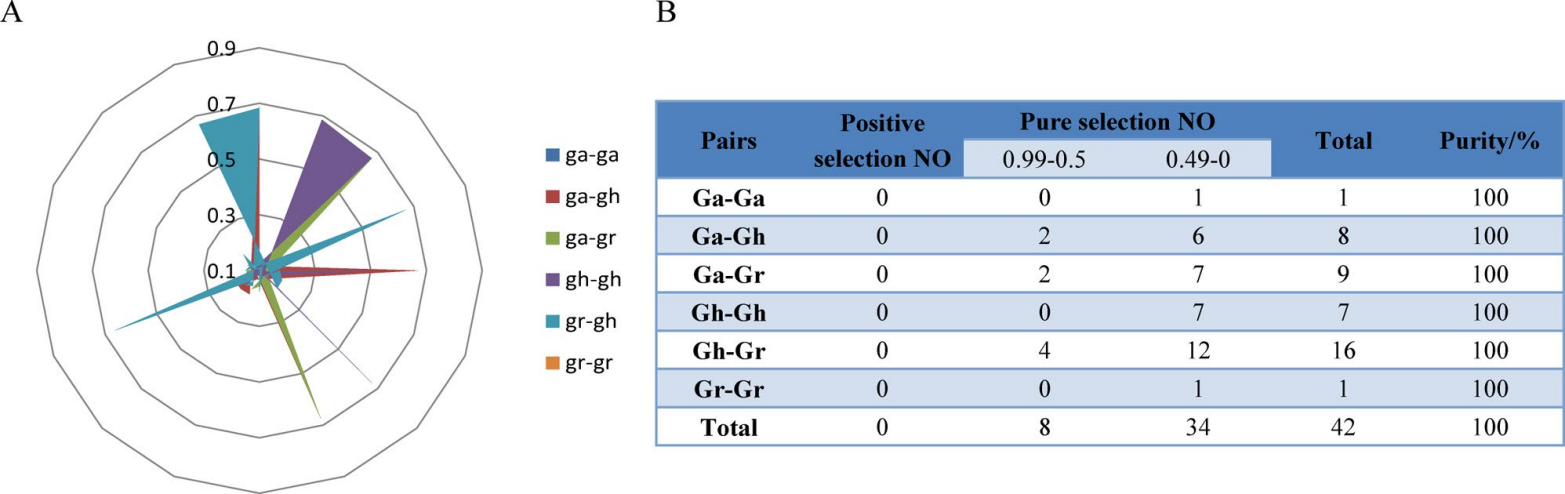
Analysis of non-synonymous (Ka) to synonymous (Ks) ratio. (**A**) non-synonymous (Ka) and synonymous (Ks) divergence values for Ga–Ga, Ga-Gr, Ga-Gh, Gh-Gh Gh-Gr and Gr-Gr are shown in circular chart. (**B**) Predicted logarithm of genes

Genomes are selected to response to the environment pressure as species evolve. For purpose of understanding the selection process of the *C5-MTase* gene family during evolution, selection stress analysis of the three *C5-MTase* genomes were performed. The ratio between no-nsynonymous (Ka) and synonymous (Ks) was calculated using software (Fig. 6A). It was considered that there was a positive selection effect when Ka/Ks was greater than 1, a neutral selection occurs when equal 1, and a negative selection effect (purification effect or purification selection) when less than 1.The results showed that there were 34 gene pairs with a KA/Ks ratio less than 0.5 for *C5-MTase*. The Ka/Ks ratio of 8 pairs was greater than 0.5 but less than 0.99(Fig. 6B). The results strongly proved that the *C5-MTases* had negative selection effect during evolution and experienced strong purification selection.

### Analysis of inter-species evolution

**Fig. 7:**
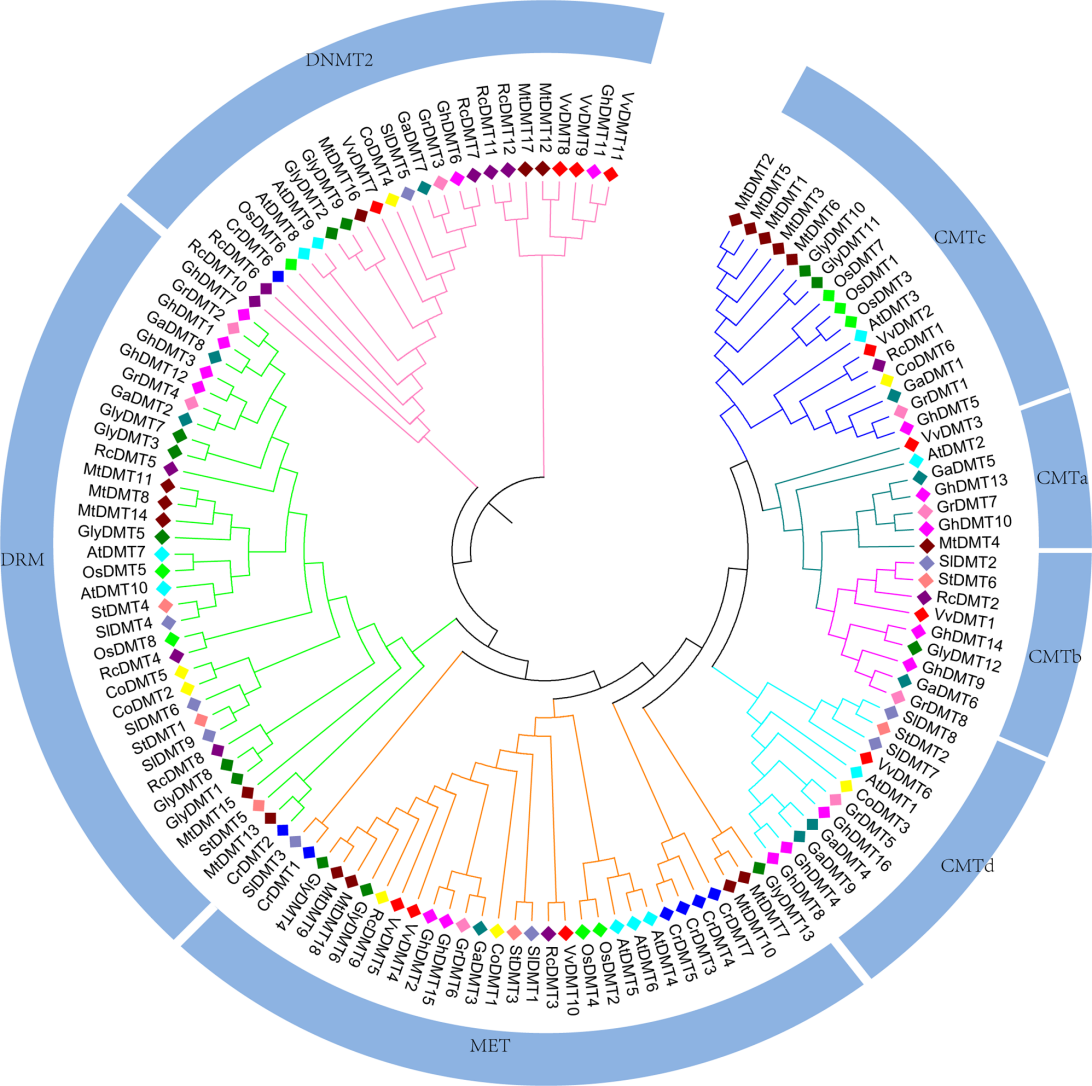
Phylogenetic analysis of *C5-MTase* family from among species. *Ricinus communis*(Rc), *Vitis vinifera L* (Vv), *Oryza sativa*(Os), *Cacao*(Co), *Chlamydomonas reinhardtii*(Cr), *Medicago truncatula*(Mt), *Arabidopsis thaliana*(At) *Solanum lycopersicum* (Sl) *Solanum tuberosum* (St) and *Glycine max* (Gly)

Phylogenetic trees can reveal the homologous and evolutionary relationships among different species. In order to investigate the evolutionary relationship between the members of the cotton *C5-MTases* family and those of *arabidopsis thaliana*, *cocoa*, *glycine max*, *oryza sativa*, *chlamydomonas reinhardtii* and other crops, the biomolecular structure of *C5-MTases* in 13 species were compared to construct a phylogenetic tree (Fig. 7). As with other crop divisions, the *C5-MTases* members of the cotton genome were divided into four subfamilies, CMT, DRM, MET, and DNMT2. Among these subfamilies, the CMT had the largest number of genes, which was divided into CMTa, CMTb, CMTc, and CMTd. There was only CMTc types existed in the monocots but more than two types of CMT existed in the dicotyledones. This suggested that the differentiation of the CMT subfamily may occur after cotyledon and dicotyledones evolution. The CMT subfamily plays an important role in the development of vascular plant, while there was no CMT in *chlamydomonas reinhardtii*, which was suspected that the CMT subfamily genes were unique to vascular plant. Interestingly, it was found that *GhDMT* numbers were lower in some subfamilies of the tetraploid species AD (*G.hirsutum*) than in *AtDMT*. For example, the MET subfamily in *arabidopsis thaliana* consisted of 3 members, while AD had only 2. Combined with this phenomenon, i.e. was speculated that *GhDMTs* in the MET subfamily played a greater important role in cotton than *AtDMTs* in Arabidopsis thaliana. DNMT2 subfamily members were present in all 13 species, indicating that the DNMT2 subfamily was highly conserved and did not differentiate during species evolution. *Cocoa* was closely related to cotton in the evolutionary branch, indicating that the *C5-MTases* in cotton was closely related to that in *cacao*.

### Expression analysis

Gene function is closed related with its expression.

**Fig. 8:**
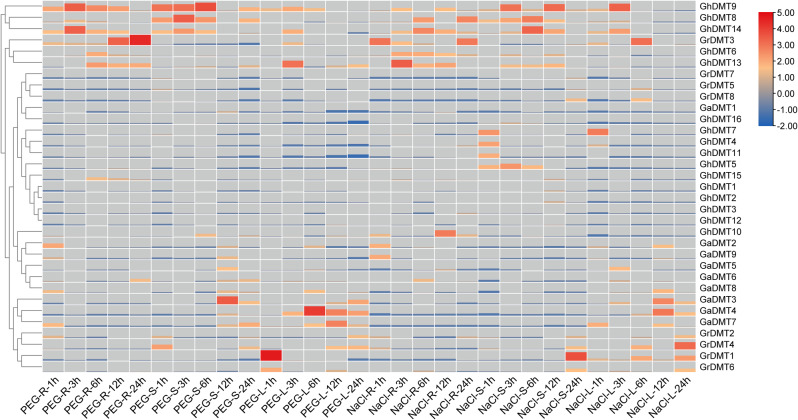
Expression analysis of *C5-MTases* from cotton under drought and salt stress

To study the expression patterns of *C5-MTases* in different cotton tissues under salt and drought stress, the *raimondii*, *Shixiya* 1, and TM-1 were developed to the trefoil stage, and real-time quantitative PCR was performed (Fig. 8). The results showed that the three cotton species had different expression patterns under different stress conditions. Through cluster analysis, it was found that *GrDMT3*, *GhDMT6*, *GhDMT8*, *GhDMT9*, and *GhDMT14* had higher expression in response to salt and drought stress. Because of *GhDMT8*, *GhDMT9*, and *GhDMT14* belong to the CMT subfamily, it is speculated that CMTa, CMTb, CMTc, and CMTd subfamily regulated a certain type of genes and played a role together in response to drought and salt stress. *Raimondii*, *Shixiya* 1, and TM-1 had obvious tissue differences when treated with drought and salt stress. *Raimondii* and Shixiya 1 had more genes up-regulated in leaf parts than in rhizome parts, TM-1 had more genes down-regulated in leaf parts than in rhizome parts. However, the responses time to stress of *C5-MTases* in *raimondii, Shixiya 1*, and TM-1 were kept in consistent. The expression level of *C5-MTases* within 12 h under drought or salt stress was high, but it was low within 12 to 24 h.

### Interaction network of GhDMT6 protein analysis

**Fig. 9:**
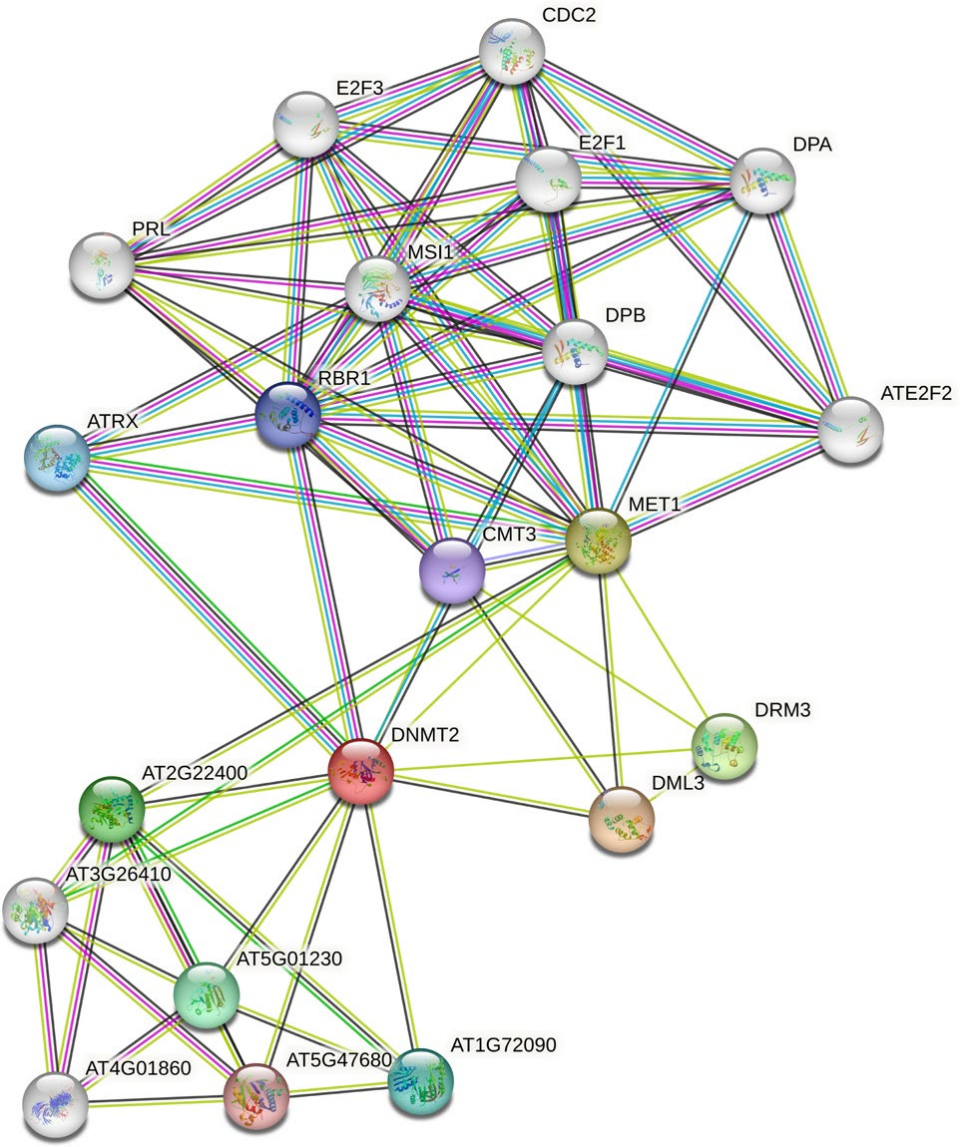
Interaction network of GhDMT6 protein

The qRT-PCRassays results showed that the *GhDMT6* was highly expressed from TM-1 under drought and salt stress. *GhDMT6* is a gene of the DNMT2 subfamily. To investigate the function of the DNMT2 subfamily, protein comparison was used to obtain orthologs of GhDMT6 in *arabidopsis thaliana*. Interaction network analysis of GhDMT6 was performed with proteins by sequence program using STRING data (Fig. 9). The function of the DNMT2 subfamily could be inferred from the in-depth study of AtDMT. GhDMT6 interacted with Retinoblastoma-related protein 1(RBR1) and Transcription factor-like protein DPB (DPB). MET1 gene interacts with member of the E2F transcription factors (E2F1, ATE2F2, E2F3). RBR1, DPB, E2F1, ATE2F2 and E2F3 were all genes related to cell division. It is inferred that *C5-MTase* is involved in cell division.

### Subcellular localization analysis of GhDMT6

**Fig. 10:**
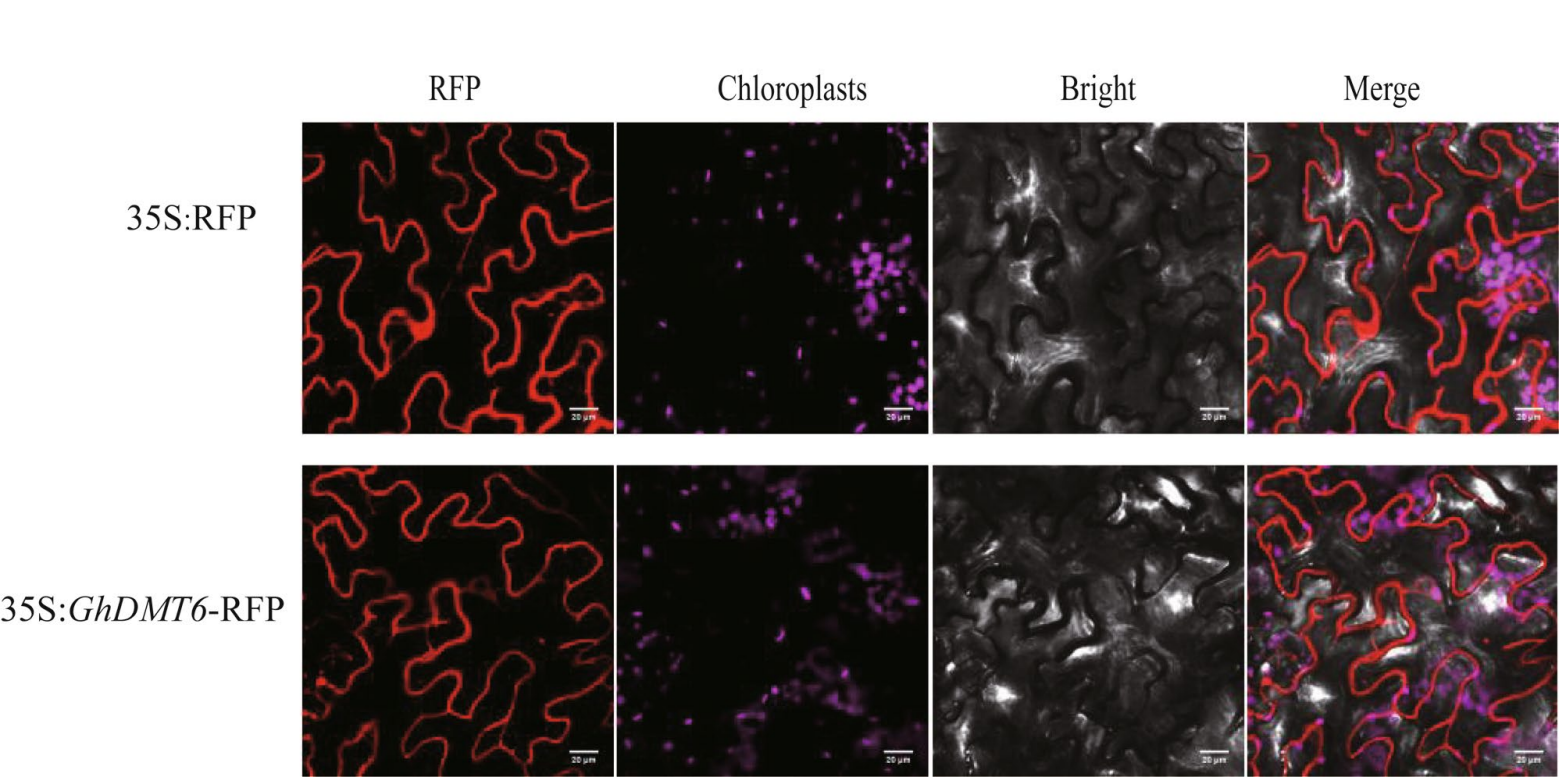
Subcellular localization of GhDMT6 protein

To further explore the function of *GhDMT6*, subcellular localization was used to determine the GhDMT6 protein location. The location of red fluorescence was observed after *GhDMT6*-RFP vector was injected into tobacco leaves. The result showed GhDMT6 protein located in the cell membrane and endoplasmic reticulum. Fluorescence of *GhDMT6* found by confocal microscopy indicated that it was mainly distributed within the cell and formed a hollow ring enclosing the nucleus with characteristic endoplasmic reticulum expression. This result demonstrated that GhDMT6 may be localized to the ER (Fig. 10).

### Expression and silencing analysis of *GhDMT6* under drought and salt stress in cotton

**Fig. 11:**
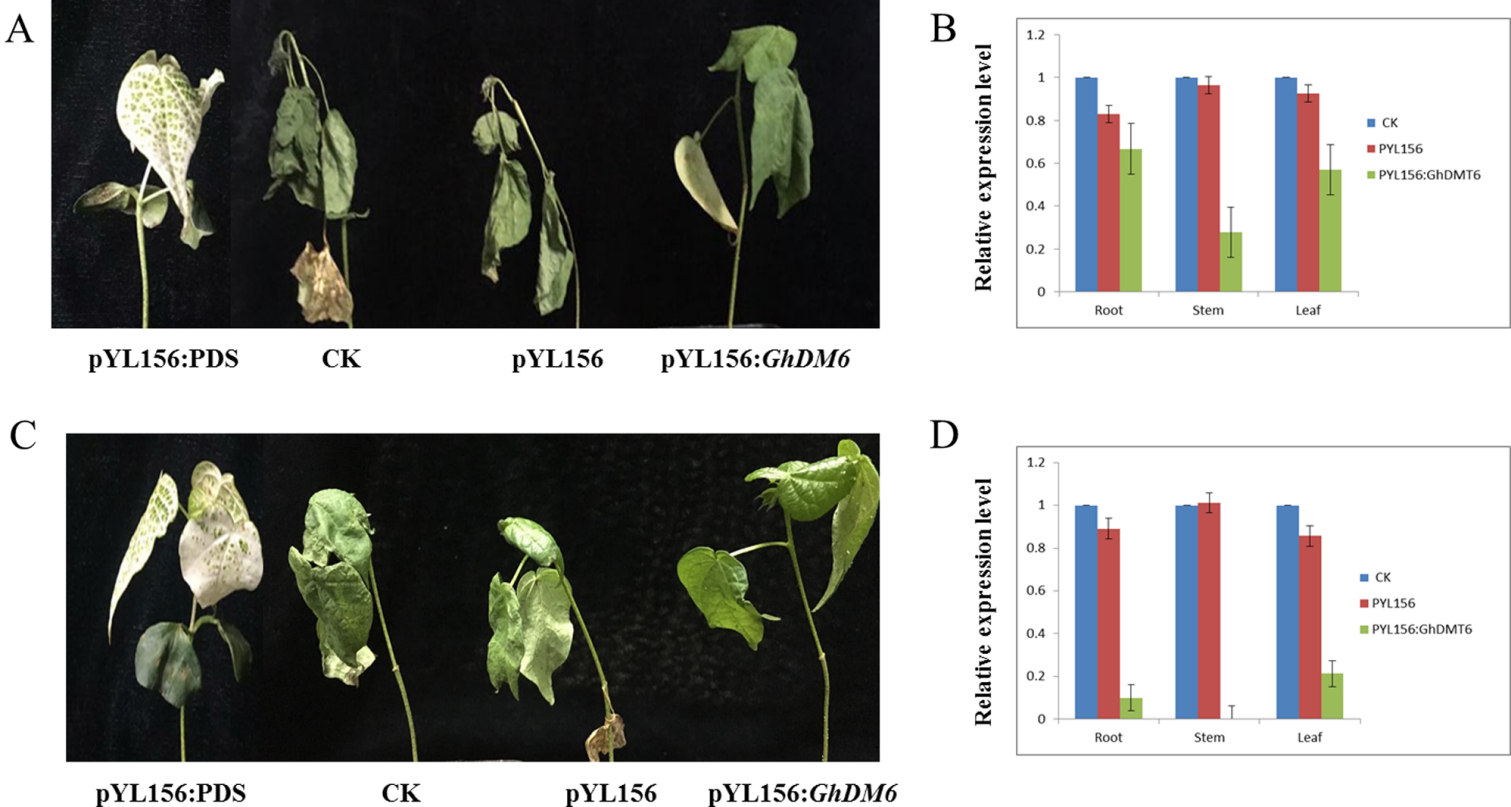
Specifically silencing *GhDMT6* gene. **A** Phenotypic differences of cotton under drought stress. **B** Expression of *GhDMT6* after drought stress. **C** Phenotypic differences of cotton after salt stress. D Expression of *GhDMT6* after salt stress

To understand the role of *GhDMT6* in response to drought and salt stress, phenotypic changes in cotton were observed by specifically silencing the *GhDMT6* gene. The leaf bleaching illustrated that the *GhDMT6* gene was specifically silenced. The fluorescent quantitative analysis showed the expression levels of *GhDMT6* in roots, stems and leaves of plants injected with pYL156:*GhDMT6* were significantly lower than control (Fig. 11B and D). After 6 days of natural drought, there were significant differences in the phenotypes of cotton seedlings. The cotyledons of CK and pYL156 seedlings were shed, and the new true leaves were yellow, withered and severely dehydrated (Fig. 11A), but the symptoms of pYL156:*GhDMT6* seedlings were milder. Similarly, after 2 days of 200 mM NaCl stress treatment, there was also significant difference in the phenotype of cotton seedlings. CK and pYL156 seedlings had shed leaves and yellowed true leaf margins, while pYL156: *GhDMT6* cotton had milder symptoms (Fig. 11C).

## Discussion

Cotton is one of the pioneer plants in saline-alkali lands because of its unique ability to resist salt and drought [36]. After the completion of the genome project of cotton [37], the identification and study of gene family classifications, evolutionary features and function prediction at the whole-genome level becomes a hotspot of cotton functional gene research, which also provides a method to study the mechanism of stress tolerance in cotton for us. Many reports have shown that DNA methylation is a response to biological adversity and regulates the expression of downstream genes on the basis of maintaining its genome unchanged [38, 39]. The methylation level in plants is a dynamic process, which initiates different genes downstream and then participates in different regulatory pathways [40]. *C5-MTases* are key enzymes in DNA methylation, which is closely related to resistance to stress [41]. However, studies of the *C5-MTases* in cotton have been largely absent. Therefore, the study of genome-wide *C5-MTases* in cotton is strongly necessary, which would have great significance of cotton breeding, the identification of functional genes and the mechanism of cotton resistance.

In this study, the classification of *C5-MTases* in different crops were quantitatively compared, which indicated that the number of DNMT2 family genes showed a big difference. *G.hirsutum* (tetraploid) had only one gene, while *arabidopsis thaliana* (diploid) had two genes. DNMT2 family members decreased as the genome increases, which may be due to the functional differentiation or loss of the DNMT2 family during evolution. The gene structure of the CMT subfamily was similar to the MET subfamily, except that there was a conserved region (chromodomain) of approximately 60 amino acid residues between motif 3 and motif 4 of the CMT gene. There was no CMTb subfamily gene in *arabidopsis thaliana*, but there were 3 genes in the MET subfamily in *arabidopsis thaliana* which were significantly more than in cotton. We infer that CMT and MET have the same or synergetic effect. The above results are consistent with the conjecture that the CMT subfamily may originate from the MET subfamily which is the closest to CMT subfamily [42, 43]. The N-terminal of CMT subfamily and MET subfamily both have BAH domains, but CMT has only one BAH domain while MET1 has two. In *arabidopsis thaliana*, the function of *AtCMT1* is lost after the BAH domain of *AtCMT1* is mutated or destroyed, and arabidopsis thaliana, CMT subfamily to maintain its own function [44].

DNA methylation in the non-CG context is widespread in the plant kingdom. Non-CG methylation in *arabidopsis thaliana* is coordinately regulated by DRM subfamily and CMT subfamily proteins. CMT may play an important role during the abiotic stress response via non-CG methylation. It was reported that *OsCMT3b* was subfunctionalized to accommodate a distinct cluster of non-CG-methylated sites at highly GC-rich regions in the rice genome [45]. Chen Zhu et al. found that *CsCMT1* and *CsCMT2* genes in *camellia sinensis* were up-regulation under drought stress and cold stress [46]. Vijay gahlaut found that *TaCMT3-6B* in *triticum aestivum* showed up-regulation when subjected to drought stress and heat stress [42]. In this study, the expression of *C5-MTases* in cotton under drought and salt stress was analyzed and it was found that CMT subfamily (*GhDMT8*, *GhDMT9* and *GhDMT14*) were up-regulated after drought and salt stress. Different from *G.hirsutum*, the DRM family members, *GrDMT2* and *GrDMT4*, were highly expressed in *G.raimondii* under drought and salt stress. It was hypothesized that non-CG site methylation occurred mainly in response to drought and salt stress in *G.raimondii*. DNMT2 is the original *C5-MTases* family [47]. How DNMT2 subfamily members play a role in response to abiotic stresses is unknown. Although the structure of DNMT2 is highly similar to the other C5-MTases, DNMT2 has only a C-terminal catalytic region and no complete N-terminal regulatory region. It has long been thought that the *C5-MTases* activity of DNMT2 may be very weak. Interestingly, the DNMT2 members, *GhDMT6*, *GrDMT3*, and *GaDMT7*, were up-regulated after PEG6000 and 200mM NaCl treatment in our study. These results indicated that DNMT2 subfamily might play an important role during the abiotic stress response via DNA methylation. It will be worth in-depth study to test this hypothesis in the future by genetic.

The Ka/Ks ratio for a total of 42 gene pairs were calculated to analyze the effects on *C5-MTases* during species evolution. It was found that the Ka/Ks ratio for all 42 gene pairs was less than 1, indicating that the *C5-MTases* family in the three cotton genomes underwent strong purification selection. In addition, this study predicted that 56 gene pairs played an important role in the expansion of *C5-MTases* during evolution (Supplemental table S1). There were fewer repetitions between Ga (A)-Ga (A), Gr (D)-Gr (D), Gh (AD)-Gh (AD), but more between Ga (A)-Gh (AD), Ga (A)-Gr (D), Gr (D)-Gh (AD). These results further confirmed that *G.hirsutum* (AD) derived from interspecific hybridization between *G.arboreum* (A) and *G.raimondii* (D) [48, 49]. In addition, we found 3 tandem repeat gene pairs that promote the evolution of *C5-MTases* function. During the evolution of cotton, the differentially expressed repetitive genes may undergo functional differentiation, and the function of *C5-MTases* can be stably preserved, indicating that the *C5-MTases* family plays a crucial role in the growth of cotton.

The promoters of *C5-MTases* in three cotton genomesand the protein-protein interaction of *C5-MTases* were detailedly analyzed. The *C5-MTase* promoter regions in three cotton species identified four stress responsiveness elements, such as defense and stress responsiveness, low-temperature responsiveness, anaerobic induction, and drought-inducibility. Most of the *C5-MTase* promoters identified plant hormone regulatory elements, such as abscisic acid responsiveness、MeJA-responsiveness、gibberellin-responsive element、salicylic acid responsiveness and auxin-responsive element, The promoter of *C5-MTase* has also found circadian control and light responsive element. All these findings demonstrated that *C5-MTase* participated in multiple signaling pathways and played an important role on defense responses, growth and development in cotton. In present study, several cell cycle genes were proved that interacted with GhDMTs protein, such as RBR1, DPB, E2F1, ATE2F2 and E2F3 [50–52]. Previous studies have shown that the cyclin-dependent kinase (CDK)-RB-E2F regulatory axis constitutes the core transcription mechanism that drives cell cycle progression, determines the timing and accuracy of genome replication, and ensures the accurate delivery of genetic material [53]. The related components regulating the CDK-RB-E2F pathway have been identified in almost all the human malignancies [54]. So GhDMTs are considered as key factor involved in cell cycle process. The growth stress mechanism of GhDMTs under abiotic stress needs further study.

Cell division, differentiation and apoptosis in plants are dynamic equilibrium processes. When plants are stressed, this balance is broken, establishing a new balance in response to stress [55]. The cell cycle is an important part of this rebalancing process. The cell cycle of eukaryotic cells refers to the cycle of continuous division of eukaryotic cells from the end of secondary mitosis to the end of the next division [56, 57]. Cell cycle is not a fixed valueor individual state, but cell cycle length is different, which reflects the state of the cell [58]. The length of cell cycle is mainly determined by the G1 phase of interphase, and the time is affected by both the organism itself and the environment [59, 60]. E2F plays an important role in regulating the cell cycle. E2F transcription factor is a member of the cell cycle gene, a key component of cyclin and retinoblastoma E2F pathway [61, 62]. Transcription factor-like protein DPB can be involved in regulating G1/S conversion. It can improve the DNA-binding activity of E2F protein after heterodimerization [63]. After the *GhDMT6* was silenced under drought and salt stress, the activity of DPB gene was enhanced and the DNA binding activity of E2F protein after heterodimerization was increased to inhibit cell division. Cell division requires a lot of nutrients and energy reserves [64]. Therefore, *GhDMT6* silenced plants showed stronger drought and salt tolerance than the control. At the same time, compared with CK plants, *GhDMT6* silenced plants after stress enhanced their resistance by prolonging cell cycle, reducing the number of cell division, and further reducing the loss of nutrition and energy, resist coercion.

## Conclusion

Based on the analysis of *C5-MTase* family in three cotton genomes, the characteristics of *C5-MTase* family were studied. In addition, we silenced the *GhDMT6*, and studied the expression of *GhDMT6* under drought and salt stress. *GhDMT6* gene is very conservative in the evolution of species. After silencing *GhDMT6* by VIGS, cotton seedlings showed enhanced stress resistance. These results provide a basis for further studies on the response of *C5-MTase* gene to abiotic stresses by delaying the number of cell divisions during plant development, which would have great significance of great significance to cotton breeding, the identification of functional genes and the mechanism of cotton resistance.

## Methods

### Identification of *C5-MTase* family in cotton

The cotton genome information was downloaded from COTTONGEN (https://www.cottongen.org/) [65]. The DNA-methylase structure domain (PF00145) was downloaded from the Pfam (http://pfam.xfam.org/) database [66] (IPR001525). The DNA-methylase.hmm hidden markov model was constructed with DNA-methylase.hmm as the reference HMMER3.0 (http://hmmer.org/ download.html). The cotton genome database was queried to obtain the gene location and name of candidate protein family members containing DNA-methylase structure domains in cotton and to obtain GFF (general feature format) files from genome annotation files. Then, the gene position was obtained on the chromosome and used local BLAST 2.2.31 + to obtain the CDS sequence and protein sequence of the corresponding gene and obtained the whole sequence of the gene corresponding to the genome based on its position on the chromosome. The gene protein sequence was downloaded in the SMART software(http://smart.embl-heidelberg.de/). The Pfam30.0 database was analyzed to ensure that each candidate gene contains a DNA-methylase structure domain. Subcellular location prediction was performed on cello [67](http://cello.life.nctu.edu.tw/). The ProtParam(https://web.expasy.org/protparam/) was obtained by protein analysis.

### Chromosomal locations of *C5-MTases* from cotton

The annotated and genomic files of the three cotton genomes were downloaded from COTTONGEN (https://www.cottongen.org/). TBtools software [68, 69] was used to map the positions of *C5-MTases* on chromosomes in three cotton genomes.

### Analysis of conserved motifs and gene structure of C5-MTases in cotton

The protein sequence of C5-MTases in cotton was input into MEME website (http://meme-suite.org/) for conservative sequence recognition and obtains MAST profile. Gene sequences and genomic sequences were entered for *C5-MTases* at the Gene Structure Display Server 2.0 website (http://gsds.gao-lab.org/) site for intron and exon analysis to obtain the tab profile. Enter the obtained files into TBtools software to draw composite images [68, 69].

### Analysis of structural domain and promoter regions

NCBI-CDD website (https://www.ncbi.nlm.nih.gov/Structure/bwrpsb/bwrpsb.cgi) was use to identify the domain contained in *C5-MTases*. DNA sequence of 2000 bp upstream of *C5-MTases* was extracted as promoter sequence. The *C5-MTase* promoters sequence was entered into PlantCare website [70] (http://bioinformatics.psb.ugent.be/webtools/plantcare/html/) for analysis to predict which cis-elements contained in the *C5-MTases* promoter.

### Collinearity and selective pressure calculation analysis

The genomic sequences and GFF files of the three cotton genomes were input into MCScanX software [71] to conduct the same-line analysis among the duplicated gene pairs from *G.arboreum*(A), *G.raimondii*(D) and *G.hirsutum*(AD). The homologous gene information was entered into the Kaks_Calculator 2.0 program for selection stress analysis [72].

### Phylogenetic analysis

Cotton DNA-methylase (PF00145, IPR001525) was used as the key word in Phytozome v12.1 [73] (https://phytozome.jgi.doe.gov/pz/portal.html) in the database rather than the homologous sequences of other species (supplementary table S2). Clustal W software was used to analyze the amino acid sequence alignment. MEGA7.0 software [74] was used to construct the phylogenetic tree with the neighbor-joining method. The number of bootstraps was 1000.

### Expression nalysis of cotton *C5-MTase* under stresses

The phytotron sand culture cultivation method was used for planting three species of cotton, TM-1 (AD), *raimondii* (D)and *Shixiya 1* (A) under16h light/8 h dark, day 28 °C, night 25 °C. Cotton seedlings were dealing with salt (200mM NaCl) and drought (20% PEG6000) stress at the three-leaf stage. The root, stem, and leaf samples of cotton were taken at 0 h, 1 h, 3 h, 6 h, 12 h and 24 h after experimental treatment. Total RNA was extracted from root, stem, and leaf samples by RN38 EASYspin Plus rapid plant RNA extraction kit and reversed transcribed into cDNA by TransScript II All-in-One First-Strand cDNA Synthesis SuperMix for qPCR (One-Step gDNA Removal) kit. The primers for the real-time fluorogenic quantitative PCR were designed with the NCBI-line primer design tool primer-BLAST (https://www.ncbi.nlm.nih.gov/tools/primer-blast/) (supplementary table S3). The RNA was reverse-transcribed into cDNA samples and used as a template for quantitative PCR experiments. QRT-PCRassays were performed on the Bio-Rad 7500 rapid fluorescence quantitative PCR platform, and the gene expression level of *C5-MTase* was detected by 2^−ΔΔCt^ method.

### Interaction network of GhDMT proteins

GhDMT6 protein sequence was used as target sequence to obtain *arabidopsis thaliana* homologous gene. The homologous gene sequence of *arabidopsis thaliana* was input into STRING software (https://string-db.org/) to further analyze the interaction between GhDMT proteins. The confidence parameter was 0.15 thresholds.

### Subcellular localization analysis of *GhDMT6*

*GhDMT6* gene was ligated into pBI121 vector containing red fluorescent protein to construct *GhDMT6*-RFP subcellular localization vector. The positive control, no-load control and *GhDMT6*-RFP vector were transferred into *agrobacterium tumefaciens* (LBA4404). Tobacco leaves were transfected with *agrobacterium tumefaciens* at 6 weeks after incubation in an indoor growth chamber, and instantaneously transformed tobacco leaves were used to visualize and locate RFP proteins under confocal laser scanning microscopy [75].

### Virus‑induced gene silencing (VIGS) of *GhDMT6*

A 423 bp DNA fragment was extracted from *GhDMT6* gene by restriction enzyme (EcoRI and XmaI) and ligated to the pYL156 vector to construct the pYL156: *GhDMT6* vector. TM-1 was planted in an artificial climate incubator and prepared the injection when the two cotyledons were flattened. pYL156:*GhDMT6*, pYL156 Vector, pYL156:PDS were injected into cotton cotyledons and continued to culture cotton seedlings until the albino phenotype appeared, indicating successful gene silencing. Cotton seedlings were subjected to drought (natural drought) and salt stress (200 mM NaCl/24 h), and observed the changes of cotton phenotype. The roots, stems and leaves of plants were took as samples, and further analyzed the relative expression levels of *GhDMT6*.

### Electronic supplementary material

Below is the link to the electronic supplementary material.

Supplementary Material 1

Supplementary Material 2

Supplementary Material 3

## Data Availability

All data supporting the conclusions of this article are provided within the article and its additional files.
