# Supplementary material for: Genome-wide characterization of DNA methyltransferase family genes implies GhDMT6 improving tolerance of salt and drought on cotton
Source: BMC Plant Biol. 2024 Apr 23;24:312. doi: 10.1186/s12870-024-04985-x (PMC11036760; doi:10.1186/s12870-024-04985-x)
Supplement: Supplementary file 1 — Supplementary Material 1 [file 12870_2024_4985_MOESM1_ESM.docx]

Supplemental table S1 Collinear gene pairs between different genomes

| **genome** | **gene1** | **gene2** |
| --- | --- | --- |
| Ga-Ga | Ga04G1492 | Ga09G0343 |
| Ga-Gh | CotAD_37635 | Ga04G1492 |
|  | CotAD_37635 | Ga09G0343 |
|  | CotAD_51709 | Ga04G1657 |
|  | CotAD_46796 | Ga04G1492 |
|  | CotAD_46796 | Ga09G0343 |
|  | Ga02G1310 | CotAD_49037 |
|  | Ga02G1310 | CotAD_14980 |
|  | Ga04G1492 | CotAD_13275 |
|  | Ga04G1657 | CotAD_46012 |
|  | Ga04G1492 | CotAD_18652 |
|  | Ga07G0535 | CotAD_40093 |
|  | Ga08G1800 | CotAD_00990 |
|  | Ga08G2785 | CotAD_04205 |
|  | Ga08G1800 | CotAD_41398 |
|  | Ga08G2785 | CotAD_68852 |
|  | Ga09G0343 | CotAD_13275 |
|  | Ga09G0343 | CotAD_18652 |
|  | Ga13G0623 | CotAD_24263 |
| Ga-Gr | Ga02G1310 | Gorai.002G216500 |
|  | Ga04G1492 | Gorai.006G031000 |
|  | Ga04G1492 | Gorai.012G062900 |
|  | Ga04G1657 | Gorai.012G048000 |
|  | Ga07G0535 | Gorai.001G052000 |
|  | Ga08G1800 | Gorai.004G180200 |
|  | Ga08G2785 | Gorai.004G274400 |
|  | Ga09G0343 | Gorai.006G031000 |
|  | Ga09G0343 | Gorai.012G062900 |
| Gh-Gh | CotAD_37635 | CotAD_13275 |
|  | CotAD_37635 | CotAD_18652 |
|  | CotAD_46796 | CotAD_13275 |
|  | CotAD_51709 | CotAD_46012 |
|  | CotAD_46796 | CotAD_18652 |
|  | CotAD_10542 | CotAD_40093 |
|  | CotAD_00991 | CotAD_41399 |
|  | CotAD_49037 | CotAD_14980 |
|  | CotAD_04205 | CotAD_68852 |
|  | CotAD_13275 | CotAD_18652 |
| Gr-Gh | CotAD_37635 | Gorai.006G031000 |
|  | CotAD_37635 | Gorai.012G062900 |
|  | CotAD_46796 | Gorai.006G031000 |
|  | CotAD_46796 | Gorai.012G062900 |
|  | CotAD_51709 | Gorai.012G048000 |
|  | Gorai.001G052000 | CotAD_40093 |
|  | Gorai.002G216500 | CotAD_49037 |
|  | Gorai.002G216500 | CotAD_14980 |
|  | Gorai.004G180200 | CotAD_00990 |
|  | Gorai.004G274400 | CotAD_04205 |
|  | Gorai.004G180200 | CotAD_41398 |
|  | Gorai.004G274400 | CotAD_68852 |
|  | Gorai.006G031000 | CotAD_13275 |
|  | Gorai.006G031000 | CotAD_18652 |
|  | Gorai.012G062900 | CotAD_13275 |
|  | Gorai.012G048000 | CotAD_46012 |
|  | Gorai.012G062900 | CotAD_18652 |
| Gr-Gr | Gorai.006G031000 | Gorai.012G062900 |
