# Supplementary material for: Genome-wide characterization of DNA methyltransferase family genes implies GhDMT6 improving tolerance of salt and drought on cotton
Source: BMC Plant Biol. 2024 Apr 23;24:312. doi: 10.1186/s12870-024-04985-x (PMC11036760; doi:10.1186/s12870-024-04985-x)
Supplement: Supplementary file 2 — Supplementary Material 2 [file 12870_2024_4985_MOESM2_ESM.docx]

Supplemental table S2 Homologous genes used for evolutionary trees construction in other crops

| Species | Gene name | Accession number |
| --- | --- | --- |
| Chlamydomonas reinhardtii | CrDMT1 | Cre03.g202150 |
|  | CrDMT2 | Cre03.g200750 |
|  | CrDMT3 | Cre06.g249500 |
|  | CrDMT4 | Cre06.g286650 |
|  | CrDMT5 | Cre06.g249350 |
|  | CrDMT6 | Cre12.g484600 |
|  | CrDMT7 | Cre10.g461750 |
| cacao | CoDMT1 | Thecc1EG001643t1 |
|  | CoDMT2 | Thecc1EG016907t1 |
|  | CoDMT3 | Thecc1EG006177t1 |
|  | CoDMT4 | Thecc1EG040279t3 |
|  | CoDMT5 | Thecc1EG038372t1 |
|  | CoDMT6 | Thecc1EG016644t1 |
| Medicago truncatula Mt4.0v | MtDMT1 | Medtr5g019420.1 |
|  | MtDMT2 | Medtr6g065580.1 |
|  | MtDMT3 | Medtr6g088715.1 |
|  | MtDMT4 | Medtr7g007090.1 |
|  | MtDMT5 | Medtr5g066780.1 |
|  | MtDMT6 | Medtr3g011150.1 |
|  | MtDMT7 | Medtr5g023660.1 |
|  | MtDMT8 | Medtr4g017850.1 |
|  | MtDMT9 | Medtr3g026190.1 |
|  | MtDMT10 | Medtr8g071100.1 |
|  | MtDMT11 | Medtr0806s0010.1 |
|  | MtDMT12 | Medtr6g005580.1 |
|  | MtDMT13 | Medtr5g066830.1 |
|  | MtDMT14 | Medtr2g009140.1 |
|  | MtDMT15 | Medtr1876s0010.1 |
|  | MtDMT16 | Medtr6g084890.1 |
|  | MtDMT17 | Medtr2g046020.1 |
|  | MtDMT18 | Medtr6g037900.1 |
| grapevine | VvDMT1 | GSVIVT01022685001 |
|  | VvDMT2 | GSVIVT01033464001 |
|  | VvDMT3 | GSVIVT01000263001 |
|  | VvDMT4 | GSVIVT01000262001 |
|  | VvDMT5 | GSVIVT01025386001 |
|  | VvDMT6 | GSVIVT01035808001 |
|  | VvDMT7 | GSVIVT01023370001 |
|  | VvDMT8 | GSVIVT01023383001 |
|  | VvDMT9 | GSVIVT01023152001 |
|  | VvDMT10 | GSVIVT01024446001 |
|  | VvDMT11 | GSVIVT01022824001 |
| Ricinus communis v0.1 | RcDMT1 | 29609.m000606 |
|  | RcDMT2 | 29631.m001043 |
|  | RcDMT3 | 29917.m001982 |
|  | RcDMT4 | 27666.m000043 |
|  | RcDMT5 | 29848.m004665 |
|  | RcDMT6 | 29889.m003366 |
|  | RcDMT7 | 28582.m000332 |
|  | RcDMT8 | 29983.m003308 |
|  | RcDMT9 | 29827.m002677 |
|  | RcDMT10 | 27640.m000037 |
|  | RcDMT11 | 45393.m000014 |
|  | RcDMT12 | 27464.m000055 |
| Oryza sativa v7_JGI | OsDMT1 | LOC_Os03g58400.1 |
|  | OsDMT2 | LOC_Os10g01570.1 |
|  | OsDMT3 | LOC_Os07g08500.1 |
|  | OsDMT4 | LOC_Os03g12570.1 |
|  | OsDMT5 | LOC_Os11g01810.1 |
|  | OsDMT6 | LOC_Os01g42630.1 |
|  | OsDMT7 | LOC_Os05g13780.1 |
|  | OsDMT8 | LOC_Os03g02010.4 |
|  | AtDMT1 | AT4G14140.2 |
| Arabidopsis thaliana TAIR10 | AtDMT2 | AT1G69770.1 |
|  | AtDMT3 | AT4G19020.1 |
|  | AtDMT4 | AT4G13610.1 |
|  | AtDMT5 | AT4G08990.1 |
|  | AtDMT6 | AT5G15380.1 |
|  | AtDMT7 | AT5G25480.1 |
|  | AtDMT8 | AT5G49160.1 |
|  | AtDMT9 | AT1G80740.1 |
|  | AtDMT10 | AT5G14620.1 |
| Solanum lycopersicum iTAG2.4 | SlDMT1 | Solyc11g030600.2.1 |
|  | SlDMT2 | Solyc08g005400.2.1 |
|  | SlDMT3 | Solyc00g015000.1.1 |
|  | SlDMT4 | Solyc10g078190.1.1 |
|  | SlDMT5 | Solyc08g067070.2.1 |
|  | SlDMT6 | Solyc02g062740.2.1 |
|  | SlDMT7 | Solyc01g006100.2.1 |
|  | SlDMT8 | Solyc12g100330.1.1 |
|  | SlDMT9 | Solyc04g005250.2.1 |
| Solanum tuberosum v4.03 | StDMT1 | PGSC0003DMP400003450 |
|  | StDMT2 | PGSC0003DMP400007625 |
|  | StDMT3 | PGSC0003DMP400053888 |
|  | StDMT4 | PGSC0003DMP400012732 |
|  | StDMT5 | PGSC0003DMP400027174 |
|  | StDMT6 | PGSC0003DMP400032223 |
| Glycine max Wm82.a2.v1 | GlyDMT1 | Glyma.07G233200.1 |
|  | GlyDMT2 | Glyma.08G177800.1 |
|  | GlyDMT3 | Glyma.19G006100.1 |
|  | GlyDMT4 | Glyma.06G178200.1 |
|  | GlyDMT5 | Glyma.02G035700.1 |
|  | GlyDMT6 | Glyma.04G187600.1 |
|  | GlyDMT7 | Glyma.05G005600.1 |
|  | GlyDMT8 | Glyma.11G083600.1 |
|  | GlyDMT9 | Glyma.17G038300.1 |
|  | GlyDMT10 | Glyma.01G160300.1 |
|  | GlyDMT11 | Glyma.16G103500.1 |
|  | GlyDMT12 | Glyma.01G007800.1 |
|  | GlyDMT13 | Glyma.15G054900.1 |
