# Supplementary material for: Genome-wide characterization of DNA methyltransferase family genes implies GhDMT6 improving tolerance of salt and drought on cotton
Source: BMC Plant Biol. 2024 Apr 23;24:312. doi: 10.1186/s12870-024-04985-x (PMC11036760; doi:10.1186/s12870-024-04985-x)
Supplement: Supplementary file 3 — Supplementary Material 3 [file 12870_2024_4985_MOESM3_ESM.docx]

Supplemental table S3 Primer sequence for qRT-PCR

| **Gene name** | **Accession number** | **Primer sequence** |
| --- | --- | --- |
| Actin | AY305733 | F:ATCCTCCGTCTTGACCTTG |
|  |  | R:TGTCCGTCAGGCAACTCAT |
| GaDMT1 | **Ga02G1310** | F:GGGCACTGATTCGACCAAGA |
|  |  | R:CGCTTACCACCACGAGAGAC |
| GaDMT2 | **Ga04G1492** | F:CTGCTCGGAAAAGGGGCTAT |
|  |  | R:CATGTATGGTGCGTGGAGGA |
| GaDMT3 | **Ga04G1657** | F:TTTCCCTACGACTCCTGCCT |
|  |  | R:CTGTCAACTGCCCATTTCGC |
| GaDMT4 | **Ga07G0535** | F:GTGCCTTGGGGCAAATATGG |
|  |  | R:AATGAAGCACACAGCCGTTC |
| GaDMT 5 | **Ga08G1800** | F:GGTCCGCTGGAAGGGATATG |
|  |  | R:ATCCCCACGAAGGGGTAAGA |
| GaDMT6 | **Ga08G1801** | F:GTCCTCCTTGGCTGGCATAA |
|  |  | R:ATCCTCGGCTGCCTCATTTC |
| GaDMT7 | **Ga08G2785** | F:TTACTCCGAGGGAGGTAGCC |
|  |  | R:TGGAGTAAGGGGGCAACAAC |
| GaDMT8 | **Ga09G0343** | F:CCGTGCAATTACTGTGCGAG |
|  |  | R:AACCACGAGACAGCTTCACT |
| GaDMT9 | **Ga13G0623** | F:AATTATGCCATCTCGGCCCC |
|  |  | R:GAATTTCCAGATGAGCCCGC |
| GrDMT1 | **Gorai.001G052000** | F:CGACCGTGTTTGCCACATTC |
|  |  | R:CTTCATGGCATAGTCCGGCA |
| GrDMT2 | **Gorai.002G216500** | F:TCTCCAGAAACACTCTGCCG |
|  |  | R:ACTCCTGCGTAACAAGACCG |
| GrDMT3 | **Gorai.004G180200** | F:GAAGCTGTTTTGGGTTCGGC |
|  |  | R:CTTGAGAACCCCATTCCGACA |
| GrDMT4 | **Gorai.004G180300** | F:TCAGCGGGGGACTTTGATTG |
|  |  | R:GATGTCTGTTGCTACCCGCA |
| GrDMT 5 | **Gorai.004G274400** | F:TCGGAAGACAGTGTTGGAGC |
|  |  | R:CTTCTTAGCGTTGTTGCCCG |
| GrDMT6 | **Gorai.006G031000** | F:GGGAATGTGCTCGGTCTCAA |
|  |  | R:TTCCTATCTGCCGGTGCTTG |
| GrDMT7 | **Gorai.012G062900** | F:TTCCCTACGACTCCTGCCTT |
|  |  | R:CTGCCCATTTCGCCACTAGA |
| GrDMT 8 | **Gorai.012G048000** | F:CCAAAGGTCGCGCATCAAAA |
|  |  | R:ATCCGCTTCCTCGGTCTTTG |
| GhDMT1 | **CotAD_37635** | F:AATTATGCCATCTCGGCCCC |
|  |  | R:GAATTTCCAGATGAGCCCGC |
| GhDMT2 | **CotAD_51709** | F:TTACTCCGAGGGAGGTAGCC |
|  |  | R:TGGAGTAAGGGGGCAACAAC |
| GhDMT3 | **CotAD_46796** | F:GTGCCGGACTATGCCATGAA |
|  |  | R:GACGGTCTGGTTGTGAGGTT |
| GhDMT4 | **CotAD_10542** | F:CTGCTCGGAAAAGGGGCTAT |
|  |  | R:CATGTATGGTGCGTGGAGGA |
| GhDMT5 | **CotAD_49037** | F:TCACTAACTTGCCGCCAGAA |
|  |  | R:TGAAGCATCCTCGGAACCAC |
| GhDMT6 | **CotAD_04205** | F:GCCTTCTTTACGCTCCCCAT |
|  |  | R:AACACTTCTTTGGCCCTCCC |
| GhDMT7 | **CotAD_13275** | F:TTCTGCACCTTGCCAACTGA |
|  |  | R:TAGTTGCGTGAGTTGTGCCT |
| GhDMT8 | **CotAD_24264** | F:CCCCAATTTCGTATGCGTGC |
|  |  | R:CCCAGCCTACAGTGCTCATC |
| GhDMT9 | **CotAD_00990** | F:TTCGTTAGGGAGAGCGCAAA |
|  |  | R:CCTTCGCGGAAAGAGAGAGG |
| GhDMT10 | **CotAD_00992** | F:CGAATGGGAATGATGGCAGC |
|  |  | R:GATTAGGACGAGCACCCCAA |
| GhDMT11 | **CotAD_14980** | F:GTCCTCCTTGGCTGGCATAA |
|  |  | R:ATCCTCGGCTGCCTCATTTC |
| GhDMT12 | **CotAD_18652** | F:GGGAATGTGCTCGGTCTCAA |
|  |  | R:TTCCTATCTGCCGGTGCTTG |
| GhDMT13 | **CotAD_41398** | F:CTGCTCGGAAAAGGGGCTAT |
|  |  | R:CATGTATGGTGCGTGGAGGA |
| GhDMT14 | **CotAD_41399** | F:CCCTTCGACTCCTGCCTTTT |
|  |  | R:AACCAGTTGACATTCCGCCA |
| GhDMT15 | **CotAD_46012** | F:AGGAAAGGGTGGTGATCGTG |
|  |  | R:TGCTGAAAAGGCAGGAGTCG |
| GhDMT16 | **CotAD_40093** | F:AATTATGCCATCTCGGCCCC |
|  |  | R:GAATTTCCAGATGAGCCCGC |
